# Crab vs. Mushroom: A Review of Crustacean and Fungal Chitin in Wound Treatment

**DOI:** 10.3390/md18010064

**Published:** 2020-01-18

**Authors:** Mitchell Jones, Marina Kujundzic, Sabu John, Alexander Bismarck

**Affiliations:** 1 School of Engineering, RMIT University, Bundoora East Campus, P.O. Box 71, Bundoora VIC 3083, Australia; 2 Institute of Material Chemistry and Research, Polymer and Composite Engineering (PaCE) Group, Faculty of Chemistry, University of Vienna, Währinger Straße 42, 1090 Vienna, Austria

**Keywords:** chitin, chitosan, wound treatment, derivatization, nanocomposites

## Abstract

Chitin and its derivative chitosan are popular constituents in wound-treatment technologies due to their nanoscale fibrous morphology and attractive biomedical properties that accelerate healing and reduce scarring. These abundant natural polymers found in arthropod exoskeletons and fungal cell walls affect almost every phase of the healing process, acting as hemostatic and antibacterial agents that also support cell proliferation and attachment. However, key differences exist in the structure, properties, processing, and associated polymers of fungal and arthropod chitin, affecting their respective application to wound treatment. High purity crustacean-derived chitin and chitosan have been widely investigated for wound-treatment applications, with research incorporating chemically modified chitosan derivatives and advanced nanocomposite dressings utilizing biocompatible additives, such as natural polysaccharides, mineral clays, and metal nanoparticles used to achieve excellent mechanical and biomedical properties. Conversely, fungi-derived chitin is covalently decorated with β-glucan and has received less research interest despite its mass production potential, simple extraction process, variations in chitin and associated polymer content, and the established healing properties of fungal exopolysaccharides. This review investigates the proven biomedical properties of both fungal- and crustacean-derived chitin and chitosan, their healing mechanisms, and their potential to advance modern wound-treatment methods through further research and practical application.

## 1. Introduction

Accidents or diseases resulting in skin damage are a commonplace occurrence in everyday life, making wound dressings a critical element of modern healthcare [1]. Wound dressings are typically porous in nature, with good barrier properties and oxygen permeability, and assist healing by preventing bleeding, absorbing excess exudates, keeping wounds moist, and protecting them from the environment [2,3]. However, issues with conventional dressings, such as irritation after prolonged use, poor compatibility with wounds, and a lack of effectiveness in the treatment of chronic wounds, such as severe burns, diabetic wounds, and ulcers, necessitates improved wound-dressing technologies that are biocompatible, actively accelerate healing, and exhibit antibacterial and antifungal activity [4,5]. Use of nontoxic and antibacterial biological polymeric nanofibers, such as chitin and bacterial cellulose, in wound-dressing technologies has subsequently received significant attention. Nanofibers are particularly well suited to wound-dressing research due to their high surface-area-to-volume ratio, porosity, pore size distribution, and morphology, which mirror the skin’s natural extracellular matrix, promoting cell adhesion and proliferation [3,6]. Chitin, a linear macromolecule composed of *N*-acetylglucosamine, and its derivative chitosan are among the most popular nanofibers used in wound-dressing research [1]. Naturally occurring in arthropod exoskeletons, mollusks, and fungi, chitin is one of the most abundant organic polymers on Earth and is easily extracted from these natural sources by using a mild alkaline treatment, the concentration of which can be increased if desired to produce chitosan, the deacetylated derivative of chitin [7]. Both chitin and chitosan exhibit properties beneficial to wound-dressing applications, including biocompatibility, biodegradability, hemostatic activity, healing acceleration, nontoxicity, adsorption, and anti-infection properties [1,3]. However, significant differences exist in the structure, properties, processing, and associated polymers of animal- and fungi-derived chitin, which have influenced their application in wound-dressing research, respectively. This review aims to investigate the key differences between animal and fungal chitin and the mechanisms through which chitin and chitosan assist wound healing, before examining the historical and current application of these chitin and chitosan variants to wound-dressing research. Advanced wound-dressing technologies utilizing chemically modified chitin and chitosan and nanocomposite architectures are also addressed, as well as the future potential of these chitin types in wound treatment.

## 2. Differences between Crustacean and Fungal Chitin

Chitin is one of the most abundant organic polymers on Earth, constituting the structural component of arthropod exoskeletons, mollusk radula, cephalopod endoskeletons, fungal cell walls, and fish and lissamphibian scales [8]. The largest source of chitin globally is suggested to be Zooplankton cuticles, with an estimated 379 million tons of Antarctic krill available worldwide [9,10]. However, fishing these tiny organisms is not commercially viable, and, subsequently, shellfish industry wastes, such as shrimp, crab, and lobster shells with chitin contents of 8–40%, are the main source of industrial chitin [11,12,13] (Table 1). Fungi provide an alternative source of chitin and, despite having lower chitin content than crustaceans (10–26% as a chitin-β-glucan complex), are experiencing increasing academic and commercial interest [14,15]. Unlike crustacean chitin, fungal chitin is not limited by seasonal and regional variation and does not require the aggressive acid treatment that crustacean chitin does for purification and demineralization, to remove calcium carbonate and other minerals [15,16]. It also supplements the rigid chitin structure with more pliable branched β-glucan, yielding a native nanocomposite architecture that can provide both strong and tough fiber networks when extracted [15,17].

**Figure 1:**
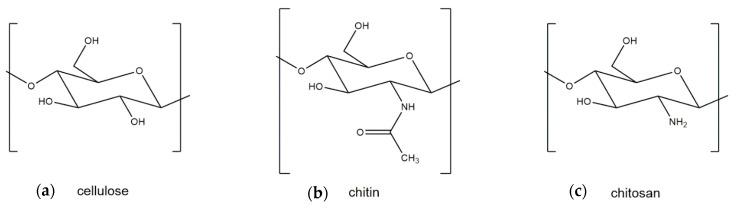
Molecular structures of (**a**) cellulose, (**b**) chitin, and (**c**) chitosan.

Both crustacean and fungal chitin have a similar molecular structure to cellulose, which is the structural component of the primary cell wall of all green plants, algae, and oomycetes. The main difference between cellulose and chitin is the replacement of the C2 hydroxyl group of cellulose with an acetamide group, which can be deacetylated to obtain chitosan (Figure 1). Two major polymorphic forms of chitin exist, α and β, with α-chitin the most common polymorph for both crustacean and fungal chitin and β-chitin occurring only in squid pen, sea tube worms, and some algae (centric diatom) [19].

However, key differences exist between crustacean and fungal chitin. Crustacean chitin normally has minimal residual protein and binds with sclerotized proteins and minerals, whereas fungal chitin is associated with other polysaccharides, such as glucan, which can occur in quantities exceeding the chitin content itself [31]. Crosslinking between chitin and proteins is well established in both crustacean- and insect-derived chitin, although it is still unclear whether the bridging is partially covalent in nature, with the low quantity of residual protein present suggesting that there is little covalent bonding or that the bonds may be cleaved during the chitin extraction process [32,33,34,35]. On the other hand, the linkages between chitin and glucan in fungi have been proven to be covalent in nature [36,37,38]. Small differences exist between insoluble glucans in mushrooms, yeast, and hyphae. However, most commonly, β-glucan exhibiting a (1→3) backbone and (1→6) branching is associated with chitin [39]. The location of chitin also varies, being concentrated in the bud scar in yeast and in the cell wall of most other fungi. Notably, in some fungi, both chitin and chitosan are simultaneously co-synthesized, a feature unique to the fungal phylum Zygomycota [40,41].

**Figure 2:**
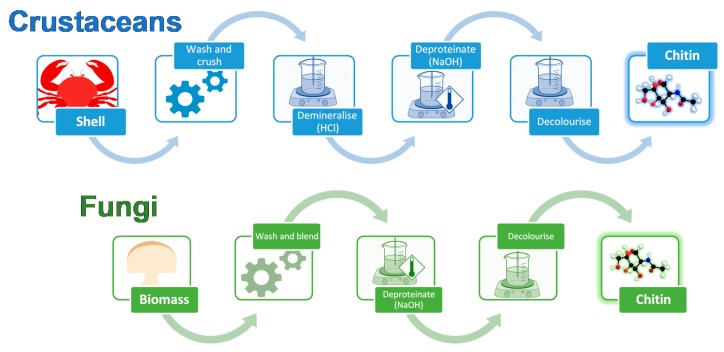
Chitin extraction process for crustacean- and fungi-derived chitin, comprising mechanical (crushing or blending) and chemical (demineralization, deproteination, and decolorization) treatments.

The extraction processes for fungal and crustacean chitin are similar (Figure 2), with both processes initially requiring the raw material to be washed and homogenized. For fungi, the starting material is mycelial biomass or fruiting bodies, while for crustaceans, it is their shells.

Fungal chitin sources are generally easily homogenized using a kitchen blender [42], while the harder and more brittle crustacean shells must be crushed. The high mineral content of crustacean shells also requires an acidic demineralization step, typically completed by using 1–2 M hydrochloric acid (HCl) for up to 48 h, although concentrations ranging up to 11 M are possible [43]. This step is not required when processing fungal chitin. Deproteination is then completed for either fungal or crustacean chitin in mild alkaline conditions, typically 1 M sodium hydroxide (NaOH), before the final material is decolorized, using a bleaching step, if desired. Pure chitin is obtained from crustacean shells as a final product, whereas fungal chitin sources yield a chitin-β-glucan complex following extraction. Pure chitin can be derived from this complex, if desired, using acid treatments to degrade the glucan, yielding X-ray diffraction patterns resembling crustacean chitin [44].

## 3. Generation and Properties of Chitosan

**Figure 3:**
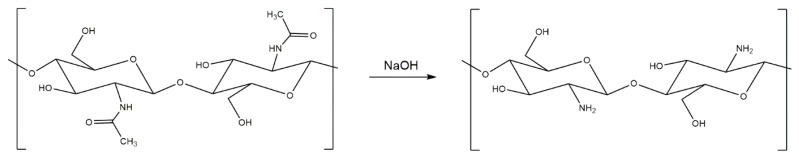
Reaction scheme for the deacetylation of chitin into chitosan, using sodium hydroxide (NaOH).

Chitosan can be generated from both fungal and crustacean chitin in a simple deacetylation process, whereby the acetyl group of chitin’s acetamide group is cleaved off under strong alkaline conditions (Figure 3), typically sodium hydroxide (NaOH), with up to 98% yields possible [45]. Although chitin and chitosan both have useful biomedical properties, including biocompatibility, biodegradability, hemostatic activity, healing acceleration, nontoxicity, adsorption, and anti-infection potential [3], chitosan generally receives more scientific attention due to its more useful structure, which renders it soluble in aqueous acids. Chitosan’s primary amine group can be protonated under mildly acidic conditions. Conversely, chitin is insoluble in all regular solvents, such as water, organic solvents, and mild acids or bases, due to the hydrogen bonding associated with the acetyl, amino, and hydroxyl groups in its polysaccharide chain [19]. Protonated chitosan’s charge also makes it a bio adhesive, able to bond to negatively charged surfaces, such as mucous membranes, chelate heavy metal ions, and is biocompatible and biodegradable with superior antibacterial properties to chitin if hydrated or in the form of a hydrogel [30,46,47]. The primary and secondary hydroxyl groups on each repeating unit and the amine group of each deacetylated unit are also reactive and are readily chemically modified to alter the physical and mechanical properties of chitosan [48]. These advantages provide chitosan with greater processing and biomedical potential than chitin as a component for wound treatment materials. However, its use in biomedical materials is limited by its poor mechanical properties. Chitin is strong, with a nanofibril tensile strength of ~1.6–3.0 GPa [49], which results from hydrogen bonding between the chains of the macromolecules [50]. Conversely, the absence of the acetyl group, which contributes to hydrogen bond formation in chitin and stabilizes its crystalline structure, significantly compromises the mechanical properties of chitosan [51]. This makes chitosan alone mechanically unsuitable for applications that require durability, such as strong films or composites, despite its significant biomedical potential.

## 4. Healing Mechanisms of Chitin and Chitosan

**Figure 4:**
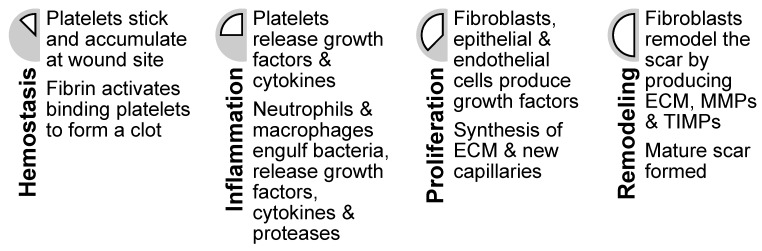
The four stages of wound healing (hemostasis, inflammation, proliferation, and remodeling) with descriptions of associated cellular activities.

Wound healing is a complex biological process comprising four stages: hemostasis, inflammation, proliferation, and remodeling [52] (Figure 4). These stages overlap in time [53] and follow a specific program, which is introduced and modulated by different cell types. Usually this mechanism works well enough to facilitate rapid repair of damaged skin. However, it does not regenerate the wounded skin completely, with scaring and loss of hair follicles or sweat glands common in healed skin [54]. Impaired wound healing function and chronic wounds in some patients is also common [52]. One of the major objectives of wound-healing technologies is to facilitate improved wound healing, tending toward wound regeneration [53,54]. Chitin and its derivates have been shown to be useful constituents in wound-dressing materials [55,56,57] and may potentially contribute to the development of skin substitutes facilitating skin regeneration since they appear to influence the wound-healing process on a molecular level.

**Figure 5:**
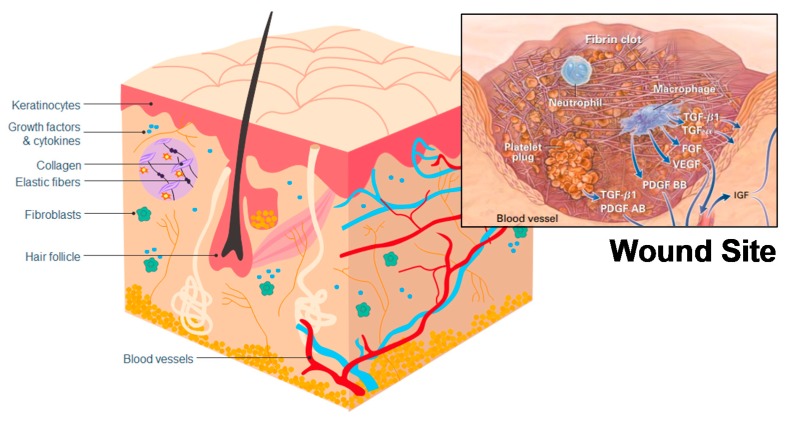
Skin structuring and locations of cells relevant to wound healing. Inset: a representation of a wound site and cells relating to the hemostasis and inflammation stages of wound healing. Reproduced with copyright permission from Singer and Clark [53].

The hemostasis phase starts immediately upon injury occurrence and is intended to stop hemorrhage by forming a fibrin clot [52] (Figure 5), a step which typically depends on platelets [54]. The formed clot re-establishes a barrier against the outside world and provides an improvised extracellular matrix, which is needed for cell migration [53]. Chitosan has hemostatic properties, reacting with red blood cells to form a coagulum [58] (Table 2), and acting independently from the regular coagulation mechanism, whereas, typically, red blood cells only have a supportive role in the formation of clots [59]. This improved hemostatic effect was also observed in whole blood, heparinized blood, and defibrinated blood [58], meaning that chitosan can potentially provide an improved clotting ability and aid hemostasis [60]. Studies have also been undertaken to assess the effectiveness of chitosan as a hemostatic in surgical settings, with wounds treated using chitosan exhibiting reduced bleeding compared to control wounds [61,62]. However, chitosan’s hemostatic effectiveness has not been compared to other hemostatics, and chitin does not seem to have been investigated as a hemostatic.

Once the hemorrhage has been stopped, the degranulating fibrin clot and surrounding tissue cells trigger the next stages of healing by releasing cytokines and growth factors, which attract cells to the wound site [53]. Chitosan may induce a different clotting mechanism, meaning that the healing process may be altered. This potentially results in a modified healing response, facilitated by the release of fewer growth factors from the platelets [63]. One of the first cells to respond next are neutrophils, also called polymorphonuclear neutrophils or PMNs. PMNs clean foreign objects, like dirt and bacteria, from the wound and remove damaged cells and are one of the main cell types responsible for inflammation [53]. Both chitin and chitosan have been shown to have a positive chemotactic effect on canine PMNs [64,65], meaning that they attract PMNs. An in vitro study showed that chitin has a stronger effect than chitosan [65]. However, chitosan may potentially impact the wound to a greater extent, as it degrades more slowly than chitin [64]. A similar effect has been observed in bovine PMNs [66]. Studies undertaken in dogs also found increased infiltration of PMNs after 3 days in wounds treated with chitosan, compared to control wounds, and decreased inflammation after 28 days in wounds treated using chitin or chitosan [67,68]. This induced increase in PMNs may also improve wound cleansing and shorten the inflammation stage, potentially providing a positive impact on the wound healing process. PMNs have also been observed to release pro-inflammatory cytokines, which may activate surrounding fibroblasts and keratinocytes [54].

Macrophages, which consume PMNs [69], become the next dominant leukocyte in the inflammation stage. Macrophages have been shown to be essential for wound healing, as they have a key role in transitioning the wound from the inflammation stage to the cell proliferation stage [52,53]. They have many functions, such as phagocytosis of dead or infected cells, attraction of many cells to the wound site, and they also support the formation of granulation tissue, blood vessels, and the extracellular matrix [53]. Defective wound repair has been observed in animals depleted of macrophages [53]. The same study, which showed increased PMN infiltration, also showed macrophages increasingly infiltrating wounds treated with chitosan in comparison to control wounds [67]. A potential reason for this may be chitosan-induced activation of a complement called C5, which attracts macrophages and PMNs [67]. Chitosan has also been shown to increase the mRNA expression and synthesis of TGF-β1 (transforming growth factor-beta 1) and PDGF (platelet-derived growth factor) in macrophages in vitro [70]. Both TGF-β1 and PDGF are chemotactic for macrophages and fibroblasts, with TGF-β1 also affecting keratinocytes, which make up the outermost layer of skin, [54] and PDGF, inducing fibroblast proliferation and collagen production [70]. It has also been observed that 70% deacylated chitin increases in vitro [71] and in vivo [72] secretion of IL-1 in macrophages, which affects fibroblast proliferation [73] and collagen production [67]. Chitosan (over 95% deacylated) on the other hand shows no effect in vitro [71] and lesser effects than the 70% deacylated chitin in vivo [72].

Fibroblasts, which lay a new skin fundament in the wound, are also a key cell type for wound healing. They produce the extracellular cell matrix [53], the structure between cells, which consists mainly of collagen [74]. Collagen plays a key role in scar formation, as excessive collagen deposition can lead to scars [53]. Insufficient collagen deposition has also been linked to chronic wounds [63]. Therefore, a balance of collagen production and degradation is necessary to ensure full regeneration. As described above, chitin and chitosan affect the secretion of different cytokines in macrophages, which in turn affect the proliferation of fibroblasts and collagen production. However, the effect of chitin and chitosan on fibroblasts is not only indirect in nature, with chitosan inducing increased IL-8 production in fibroblasts [66], a strong chemotactic for PMNs and regulator of keratinocyte migration and proliferation [73]. Another important role of fibroblasts is the production of the extracellular cell matrix. In vitro studies have shown no direct effects of chitosan on the fibroblasts producing the extracellular matrix [66]. However, indirect effects through microphage stimulation, and therefore fibroblast stimulation, may affect this stage. It has also been hypothesized that chitin and chitosan could be incorporated into the extracellular matrix, through the use of lysozyme [60], an enzyme capable of degrading chitin and chitosan.

The effects of molecular weight and degree of deacetylation on the wound-healing potential of chitin and chitosan have also been studied, albeit to a lesser extent. An in vivo study on incisions in rats investigated the effect of chitin (300 kDa, <10% deacetylation), chitosan (80 kDa, >80% deacetylation), and their oligomers and monomers on wound break strength and collagenase activity [75]. The results showed that wounds treated using chitosan (chitosan, oligomer, and monomer) were stronger than those treated using chitin. In both cases, treatments using oligomers were associated with the highest wound break strength, although chitosan had comparable performance, and chitosan monomers were associated with the highest collagenase activity. A further in vitro study on fibroblasts investigated differences in healing based on the molecular weight and degree of deacetylation of chitin and chitosan [76], utilizing chitin with a degree of deacetylation of 37% and molecular weights of 37 and 197 kDa, and chitosan with degrees of deacetylation of 58% and 89% and molecular weights of 12 kDa/194 kDa and 13 kDa/263 kDa, respectively. The results showed that chitosan with a higher degree of deacetylation had a greater effect on fibroblast proliferation, which was further enhanced at lower molecular weights.

The molecular weights of the chitin and chitosan used in these studies falls in the low-molecular-weight range, as both chitin and chitosan can have molecular weights >1000 kDa [77]. However, another in vivo study investigating surgical burns in rats used a broader range of molecular weights, ranging from 70 to 750 kDa and peaking at 2000 kDa [78]. However, the degree of deacetylation of each sample was not varied, with a fixed value of 63%, 75%, and 92%, for each respective molecular weight. The results showed that the 2000 kDa chitosan performed significantly better than the other molecular weights both in wound contraction and collagenase activity, a result that the author attributed to its high molecular weight [78]. However, since the degree of deacetylation has a greater effect on wound healing than the molecular weight in the low-molecular-weight range [75,76], the effects of different degrees of deacetylation at high molecular weights should also be examined.

It is however clear that chitosan with a higher degree of deacetylation exhibits higher biological activity than that with a lower degree of deacetylation [75,76]. The effect of molecular weight on wound healing in the low-molecular-weight range studied (<300 kDa) also seems to be enhanced at lower molecular weights (<100 kDa). However, since chitin and chitosan can exhibit molecular weights that fall well outside this range, further studies for higher molecular weights (>300 kDa) are required, especially when considering the correlation between the hemostatic activity of chitosan and higher molecular weights [79].

## 5. Fungi-Derived Chitin and Chitosan Wound Dressings

**Figure 6:**
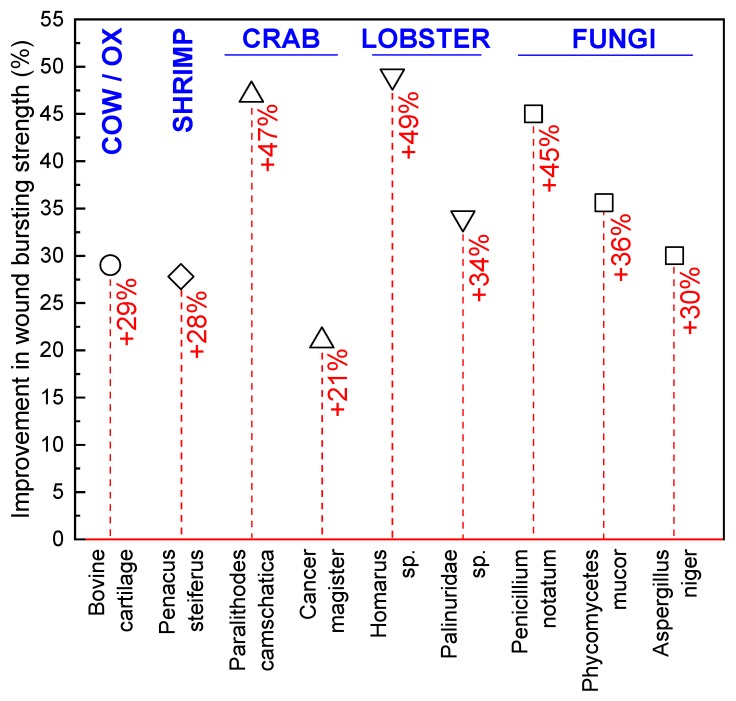
Improvement in the bursting strength (%) of wounds treated using bovine cartilage and shrimp, crab, lobster, and fungal chitin, compared to an untreated control wound. Data from Balassa and Prudden [85].

Medical applications of fungi date back to ancient times, where it was used as a styptic to stop bleeding and as a crude precursor to modern antibiotics in the treatment of infections [80,81,82,83]. However, true academic interest in medical materials derived from fungi did not begin until the 1970s, when the mycelia of several fungal species were investigated as wound-healing accelerants. Prudden et al. [84] studied the topical application of powdered mycelium from *Phycomycetes mucor*, *Penicillium notatum*, and *Aspergillus niger* on rat wounds to confirm the healing properties of glucosamine (polymer units of chitosan), which was thought to be responsible for the healing potential of the cartilage material historically used in wound treatment [84]. Wounded rat skin treated with powdered mycelium was found to have a higher tensile strength than untreated or cartilage powder treated wounds as they healed, with *P. mucor* associated with the highest skin tensile strengths (Figure 6). In further investigations, a chitin-β-glucan powder was also produced through NaOH and HCl treatment of the same mycelium and compared to purified crustacean chitin, again as a topically applied healing accelerant on rat wounds. Lobster and king crab chitin outperformed the purified mycelium powder; however, all fungal species except *A. niger* provided higher skin tensile strength than the cartilage and shrimp chitin healing agents [85].

Following these successes, chitin quickly gained popularity academically as a wound-healing accelerant, with most subsequent work focusing on the healing potential of crustacean chitin. However, the lower costs and simpler purification of fungal chitin did attract research interest, with further studies utilizing NaOH and acetic acid purified *Aspergillus oryzae*, *Mucor mucedo*, and *Phycomyces blakesleeanus* mycelium demonstrating increased cell proliferation in fibroblasts at low concentrations. This cell-proliferating effect facilitated through the use of the fungal material was found to correlate with the chitin or chitosan content of the material, with *P. blakesleeanus* showing the highest proliferation at 0.01% and 0.5% w/v. Additionally cell attractant properties were found in *P. blakesleeanus* and *M. mucedo*, which were suggested to assist in wound healing [86].

**Figure 7:**
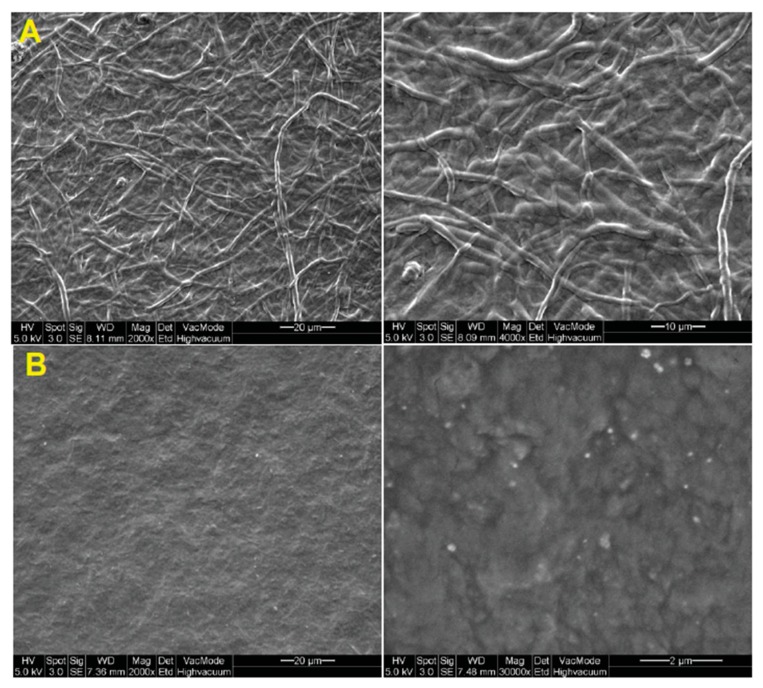
SE (scanning electron) micrographs of (**A**) fibrous and (**B**) micronized Sacchachitin. Reproduced from Chen, Lee, Chen, Ho, Lui, Sheu, and Su [88] (open access).

Commercialization of fungi-derived wound treatment materials occurred in 1997, with a research group from Taiwan extracting a chitin-polysaccharide mixture from *Ganoderma tsugae*, comprising β-1-3-glucan (~60%) and *N*-acetylglucosamine (~40%), and creating a weavable skin substitute called Sacchachitin (Figure 7). This novel wound dressing was tested on rats [56] and Guinea pigs [87], before being tested in a preliminary clinical trial on two human patients with chronic wounds, in 2005 [55]. Animal studies showed that Sacchachitin improved wound healing significantly compared to conventional gauze and had comparable performance to Beschitin, a commercially available wound dressing from crustacean chitin, developed in 1988 [87]. Improvements in healing were also observed in human trials for chronic wounds open for seven months or longer, with the underlying healing mechanisms being fibroblast and keratinocyte proliferation and the activity of matrix metalloproteinases (MMPs) (human cells) [55]. A Sacchachitin nanogel derivative was also produced for the treatment of corneal burns in rabbits, demonstrating promise with significant increases in cornea cell proliferation and wound closure stimulation, in addition to enhanced corneal wound healing due to the inhibition of protein breakdown [88].

More recently, with the rise of research interest into the wound-treatment potential of purer crustacean chitin derivatives, academic interest in the medical applications of fungal material has shifted to the investigation of fungal exopolysaccharides (EPS). EPS are not components of the fungal intercellular matrix or cell wall, where chitin is typically found, but rather occur on the cell surface or in the extracellular matrix [89]. Compared to other fungal polysaccharides, they are mass producible in a short time and easily isolated and purified [89]. Thai studies investigating the EPS of 16 different native fungal strains identified three strains that were biocompatible with Vero cells, which are primate cells resembling fibroblasts, and increased interleukin-8 (IL-8) production in fibroblasts, improving wound healing [90]. EPS have also been combined with traditional antibacterial agents, such as ciprofloxacin, to create active-agent-loaded fungi-derived wound dressings [91]. Fungal β-glucans, such as lentinan from *L. edodes* (shiitake), schizophyllan from *S. commune* (split gill), zymosan from *S. cereviase* (baker’s yeast), pleuran from *P. ostreatus* (oyster), and ganoderan from *G. lucidum* (reishii), have also been extensively studied due to the human immune system’s ability to recognize them, promoting immune stimulation, antibacterial, antitumor, anticancer, and antioxidant properties [92,93,94,95]. These findings, coupled with the varying chitin, chitosan, and polysaccharide profiles of the over 5.1 million species of fungi in existence [96] and recent advances in fungal material technology [7,14,17,97,98,99,100,101], suggest that fungi-derived wound treatments warrant further investigation. In particular, the native chitin-β-glucan composite architecture of fungal chitin could be utilized to achieve scaffolds exceeding the mechanical performance of crustacean chitin [17] and novel antibacterial properties resulting from composite dressings incorporating naturally generated complexes of fungal chitin, chitosan, β-glucans, and exopolysaccharides could pave the way for new low-cost, natural, and mass-producible dressing technologies.

## 6. Crustacean-Derived Chitin and Chitosan Wound Dressings

### 6.1. Derivatization of Chitin and Chitosan to Improve Solubility and Biomedical Properties

Most research concerning the application of crustacean-derived chitin and chitosan in wound dressings focuses on either chemical modification (derivatization) or material engineering practices, such as hybridization or incorporation of chitin or chitosan into nanocomposites, to address the physical, biomedical, or mechanical shortcomings of each respective polymer. Derivatization often deals with improving the solubility of chitin and chitosan [102], which typically have low solubility in common solvents, with chitin being insoluble in water and most organic solvents, and chitosan being insoluble in most organic solvents and aqueous solutions above pH 6.5. This hinders the processing of chitin and chitosan and limits their applications. Other goals of derivatization include the addition or enhancement of existing biomedical properties of chitin or chitosan, such as antibacterial activity [103], hydrogel formation [104,105], and wound-healing acceleration. The most common derivatizations are carboxymethylation, which introduces the carboxymethyl functional group, and quaternation, which converts a tertiary amine to a quaternary ammonium compound; however, other derivatizations are also possible.

**Figure 8:**
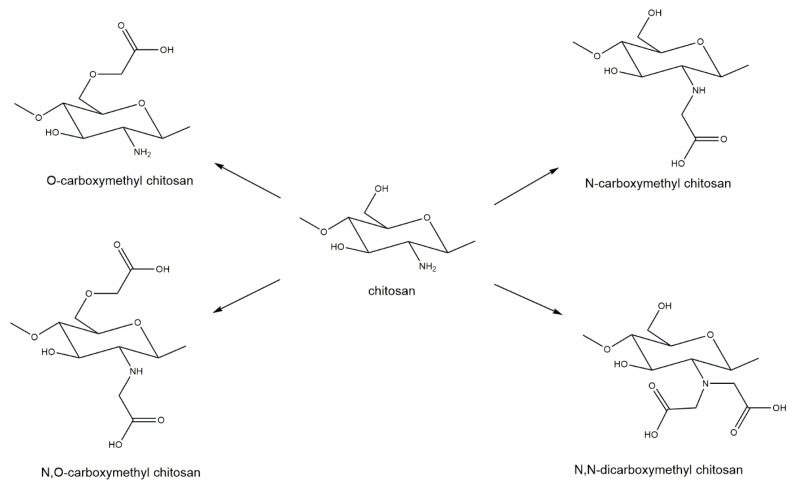
Carboxymethylation derivatizations of chitosan generating O-carboxymethyl chitosan, *N*-carboxymethyl chitosan, *N,O*-carboxymethyl chitosan, and *N,N*-dicarboxymethyl chitosan.

Carboxymethylation of chitin and chitosan is commonly regarded as one of the most useful derivatizations. Through the addition of the carboxymethyl group to chitin or chitosan, an anionic functional group (carboxyl) is introduced. This addition makes chitin or chitosan more hydrophilic and improves solubility, both in water and some organic solvents [106]. Carboxymethylation of chitin and chitosan is also known to improve biocompatibility and antibacterial properties and can be used to create hydrogels more effectively [106,107]. The specific properties of a carboxymethylated chitosan depend on the degree of carboxymethylation and the substituent position of the carboxymethyl group [108]. Four different substituent patterns for chitosan have been reported: *O*-carboxymethyl chitosan, *N*-carboxymethyl chitosan, *N,O*-carboxymethyl chitosan, and *N,N*-dicarboxymethyl chitosan [107] (Figure 8).

Although all of these variants are water-soluble, *N,O*-carboxymethyl chitosan has better antibacterial properties than *O*-carboxymethyl chitosan and unmodified chitosan [109,110]. These improved antibacterial properties are most likely the reason for the heightened research interest in *N,O*-carboxymethyl chitosan. *N*-carboxymethyl chitosan has been shown to improve wound healing in mice by inducing production of inflammatory cytokines [108] and *N,N*-dicarboxymethyl chitosan has been shown to be associated with bone regeneration by chelating calcium and magnesium [111,112]; however, both substrates have received less attention from the research community.

Notably, carboxymethylated chitin or chitosan can be further functionalized through the introduction of additional substituents to the backbone of the chitin or chitosan structure or the carboxyl group of the carboxymethyl. Some examples of further functionalization include the addition of acrylic groups, like 2-hydroxy-3-methacryloyloxypropylate, to the backbone, resulting in a tissue adhesive utilizable in wound-closure applications [113]. Ethylenediamine can also be introduced at both 3-OH and 6-OH positions in chitin or chitosan to create 3,6-*O*-*N*-acetylethylenediamine modified chitosan (AEDMCS), which has improved solubility in water over a wide pH range (3–11, compared to 3–6) and exhibits improved antibacterial properties, against both Gram-negative and Gram-positive bacteria [114]. Further functionalization of carboxymethyl chitin at the carboxyl group also provides the opportunity to produce improved hydrogels, such as those incorporating tyramine groups. The phenolic group of tyramine can be enzymatically crosslinked by using horseradish peroxides (HRP), which can be used to tune hydrogel properties [115].

Quaternization is another popular chitin- and chitosan-derivatization process, which converts a tertiary amine to a quaternary ammonium compound, enhancing solubility in water and organic solvents, as well as increasing antibacterial activity [109,116,117]. These improved properties are associated with the addition of the permanent cationic group RN(CH_3_)_3_^+^, with the increased charge generating a higher polar character in chitin or chitosan and enhancing solubility in polar solvents, like water and some organic solvents. The leading hypothesis regarding the improved antibacterial properties of these derivatives is that increases in cationic groups result in interaction with the negative surface of bacteria, which inhibits bacterial growth [109,116,118,119]. Various substituent patterns are achievable depending on how chitin or chitosan are derivatized. Chitin’s accessible position for derivatization is the 6-OH group [120,121]. However, simultaneous quaternization of the 6-OH and 3-OH positions also seems to be possible and may provide superior antifungal activity than 6-OH quaternization alone [118]. Conversely, derivatization of chitosan is most readily achieved through *N*-quaternization, as the amine group simply needs to be methylated, resulting in *N,N,N*-trimethylchitosan (TMC), which exhibits improved solubility and antibacterial properties. However, the unintended methylation of the OH-groups of chitosan can also accrue under the classical synthesis route, which may reduce the solubility of the product. Such potential methylation has led to the creation of *O*-methylation free TMC synthesis procedures [116]. Other quaternization positions in chitosan include the 6-OH group and the diquaternization of both N and O positions [119].

The mechanical properties of chitosan-based wound dressings are also sometimes the focus of derivatizations utilizing crosslinking. Despite the unsuitability of polyvinyl alcohol (PVA) and polyvinylpyrrolidone (PVP) hydrogels themselves as wound-dressing materials due to their insufficient elasticity and limited hydrophilicity [122], they are popular crosslinkers for chitosan-based hydrogels, to improve their tensile properties [123,124,125,126]. Crosslinking with genipin, a chemical compound found in gardenia fruit extract, is also popular [3,127].

Many other derivatizations of chitin and chitosan are also possible, including sulfonation, which leads to properties resembling heparin, a blood thinner [128], and phosphorylation, which increases solubility and antibacterial properties [129]. Introduction of ether groups is also possible, creating derivatives like hydroxypropyl chitosan, which has antifungal properties against fruit fungi [130], or hydroxyethyl β-chitin (HEC) and hydroxybutyl β-chitin, which are antibacterial chitin derivatives that can be turned into gels through heating [131].

### 6.2. Chitin and Chitosan Nanocomposite Architectures as Wound Dressings

Recent advances in chitin- and chitosan-based wound dressings include polymer, mineral, and metal additives, rather than utilizing pure chitin or chitosan. These additives are typically included to improve the mechanical properties, such as tensile strength and modulus, and biomedical properties, such as antibacterial and antifungal activity of the dressings [1]. However, improvements in these properties must be achieved without compromising dressing biocompatibility, such as blood compatibility and cytocompatibility, physical properties, such as porosity and surface area, which affect cell and fibroblast attachment, and wetting and barrier properties, such as hydrophilicity, water uptake capacity, oxygen, carbon dioxide, and water vapor transmission rates [124,132]. This is important since chitin and chitosan themselves are biocompatible and able to be degraded by several enzymes [133], such as lysozyme in vivo [134]. The degradation rate is governed by the molecular weight and degree of deacetylation [133,134] of the chitosan with further manipulation based on fiber diameter and mesh porosity also possible in chitosan fiber-mesh scaffolds [135].

Biocompatible additives include natural polysaccharides, such as cellulose, and mineral clays, such as the aluminum phyllosilicate clays bentonite and halloysite. These additives are primarily used as reinforcements to improve the mechanical properties of wound dressings [1], which is especially important in chitosan-based wound dressings, which suffer from poor tensile strength and elasticity [132]. Cellulose nanocrystals [127,136] increase the tensile properties of chitosan-based wound dressings, with the additional possibility of utilizing bacterial cellulose to facilitate amine coupling, to increase strength rather than conventional impregnation or physical blending methods [137]. Alternatively, chitin itself, which has a high tensile strength and modulus, can be used to reinforce chitosan, with both chitin nanofibers [132] and nanocrystals [3] improving the tensile strength of chitosan-based wound dressings. Combinations of chitin and silk fibroin are also popular in wound-dressing research [138,139,140], as are combinations of chitosan and sodium alginate, which has hemostatic and gel-forming properties, keeping the wound moist and preventing fiber entrapment during removal [141,142]. Use of these fibrous nanomaterials in wound dressings not only improves tensile strength, but also typically increases surface area, improving fibroblast attachment and spreading [143], without significantly affecting biocompatibility or barrier properties [3,124].

Minerals, such the aluminum phyllosilicate clays bentonite and halloysite, can alternatively provide increases in tensile strength [144,145], while additionally increasing glass transition temperature and enhancing some biomedical wound dressing properties. For example, bentonite clays are nontoxic, have a high cation-exchange capacity, and provide some degree of antimicrobial activity, while also being low in cost and abundant [146,147]. Bentonite is very hydrophilic and is subsequently able to absorb large amounts of wound fluid, increasing the water uptake capacity of dressings and maintaining the moist environment necessary for wound healing [148]. Halloysite nanotubes are also popular nanofillers to improve the mechanical properties of wound dressings due to their unique rod-like structure and hydrophilicity, meaning that they are easily solution-mixed with chitosan in aqueous solution, facilitating easy nanocomposite preparation [149,150,151]. Graphene oxide is also sometimes included due to its amphiphilicity, high intrinsic strength, and ample oxygen-bearing groups, in addition to its high surface area, which, like nanofibers, was found to improve fibroblast attachment and spreading [143].

**Figure 9:**
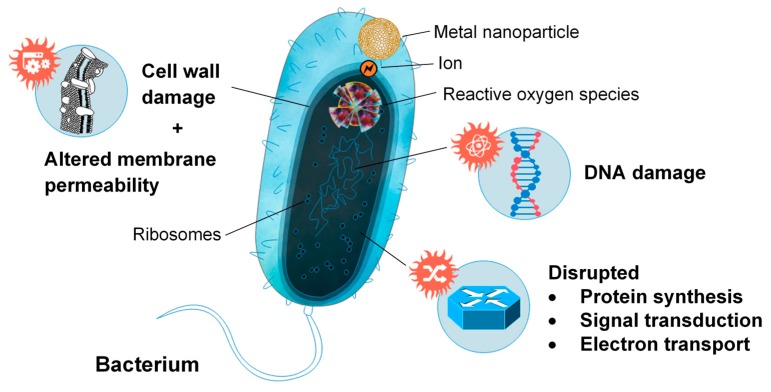
Metal nanoparticle interfacing with bacterium and representations of the mechanisms facilitating microbial death.

However, while many additives are included in wound dressings as reinforcement, others serve purely to enhance the antibacterial properties of the dressing. Metal nanoparticles are popular additives to chitosan-based wound dressings due to their ability to alter the metabolic activity of bacteria they encounter, crossing the bacterial membrane and affecting the shape and function of the cell membrane [143,152] (Figure 9). The presence of these nanoparticles in the metabolic pathway causes oxidative stress, enzyme inhibition, protein deactivation and altered membrane permeability, electrolyte balance, and gene expression, which results in microbial death [153]. Most commonly, silver (Ag), gold (Au), copper (Cu) zinc oxide (ZnO), and titanium dioxide (TiO_2_) nanoparticles were incorporated into chitosan-based wound dressings, to enhance antibacterial activity [5,126,154,155,156,157,158,159]. Ag and Au nanoparticles reduce oxygen molecules, to form reactive intermediates with strong positive redox potential, such as superoxide and hydroxyl radicals (Ag nanoparticles), and singlet oxygen (Au nanoparticles). Metal oxides, such as ZnO and TiO_2_, behave similarly, interacting with water or hydroxide ions to form hydroxyl radicals, which can be reduced to superoxide. These radicals then degrade active components governing normal bacterial morphological and physiological function, providing an antibacterial effect [153]. Similar antibiotic activity has also been investigated by using honey and antibiotic-loaded chitosan-based wound dressings, but has received less attention than wound dressings utilizing metal nanoparticles [4,152,160,161].

## 7. Human Clinical Trials Utilizing Chitin and Chitosan for Wound Treatment

Commercialization of chitin and chitosan-based wound treatment products, such as Axiostat^®^, Beschitin^®^ W, Bexident^®^ Post, Celox^®^, Chitohem^®^, HemCon^®^, Medisorb^®^ R, Surgi shield^®^, and SEQUA^®^ San Chitosan, in addition to custom-prepared treatments, have prompted several human clinical trials of chitin and chitosan for wound-healing applications. These studies predominantly focus on chitosan and chitosan derivatives for use as wound dressings, gels, powders, films, membranes, and even mouthwash for oral, nasal, ear, and skin applications, in addition to treatment of ulcers and serious hemorrhages (Table 3).

These wound treatments have experienced significant success in oral clinical trials where they have been used to treat aphthous stomatitis (mouth ulcers), postoperative and tooth extraction wounds. Chitosan mouthwash (0.5%) reduced aphthous stomatitis pain and ulcer size, exhibiting comparable performance to Triamcinolone, a corticosteroid product [165]. Similar results could also be achieved by using a mucoadhesive film [166]. Dental surgery clinical trials also found chitosan-based wound treatments to significantly shorten bleeding time and improve wound healing for patients undergoing minor surgery and tooth extractions [167,168]. Clinical trials examining the effect of chitosan on a range of other wounds also exhibited very promising results, with improved cell adherence, hemostasis and re-epithelialization [164,171], less itching and sensitivity [171], less exudate and odor [175], and reductions in wound healing time [170,173], post-hemodialysis puncture site bleeding [173], and rebleeding [174] in diabetic, puncture, superficial, skin graft, and sinus postoperative wounds. Chitosan has also been utilized in clinical trials for treatment of ulcers with notable reductions in exudate, pain on dressing removal, wound area and depth when compared to traditional Vaseline gauze dressings [176,177]. Additionally, military-grade chitosan-based wound-treatment products, such as Celox^®^, have been used as lower-cost alternatives to oxytocin, prostaglandin, and uterine balloons in the treatment of life-threatening obstetric hemorrhages, completely stopping uncontrolled bleeding within seconds to minutes. Although chitosan was used in most clinical trials, it should also be noted that chitin membranes have been used to assist in the closure of chronic tympanic membrane perforations in the ear [162].

## 8. Conclusions

Chitin and its chitosan derivative have experienced significant research interest for wound-dressing applications since the 1970s due to their ability to accelerate healing and reduce scarring. While chitin historically received academic attention due to its biomedical properties, its deacetylated derivative chitosan has been the focus of more recent scientific research due to its superior healing properties. These beneficial properties of chitin and chitosan include nontoxicity, biocompatibility, biodegradability, and antimicrobial activity and are achieved through numerous mechanisms, such as hemostatic activity and support in cellular proliferation and attachment. Crustacean-derived chitosan has been widely investigated and used in wound-healing research due to its high yields and purity, with significant advances in wound dressings incorporating chemically modified chitosan derivatives to improve solubility and antimicrobial activity. Chitosan blends incorporating biocompatible additives, such as natural polysaccharides, synthetic polymers, mineral clays, and metal nanoparticles, have also been utilized to generate advanced wound dressings with excellent mechanical and biomedical properties. Fungi, on the other hand, have received significantly less scientific interest due to their lower chitin content. Moreover, the chitin present in fungi is covalently linked to β-glucan. However, the mass production potential and simple extraction processes associated with fungal chitin, coupled with variations in the chitin content of fungal species, recent advances in fungal materials technologies, and the discovery of healing properties in fungal exopolysaccharides suggest that further investigation of the healing potential of fungal polysaccharide constituents is warranted. With proven biomedical properties, both fungi- and crustacean-derived chitin and chitosan constitute powerful natural medicinal agents, with the potential to advance modern medicine and wound treatment through further research and practical application.

## Figures and Tables

**Table 1:** Polymorphs and examples of chitin sources with their respective chitinous constituent dry weight (d.wt.) compared to total source mass, chitin contents, and other major organic and inorganic constituents listed. Data from [14,18,19,20,21,22,23,24,25,26,27,28,29,30].

Polymorph	Sources	Chitin Content	Other Major Constituents
α	Crustacean shells	(chitinous shell up to 50% of crustacean d.wt.)	
	Lobster	16–23%	20–60% calcium or magnesium carbonate, 20–40% protein
	Crab	25–30%	
	Krill	34–49%	
	Insect cuticles	(chitinous cuticle up to 50% of insect d.wt.)	
	Cockroach	18–38%	20–50% protein, minerals, pigments and fat
	Butterfly	22–64%	
	Silkworm	20–44%	
	Fungal cell walls	(chitin–glucan nanofibers up to 26% of fungal biomass d.wt.)	
	Mushrooms	8–43%	50–60% β-glucan, protein
	Mycelium	5–35%	
	Yeast	1–3%	
	Mold	8–27%	
β	Squid pen	31–49%	Proteins and minerals
	Sea tube worms	25–29%	

**Table 2:** Cell types, their respective functions in wound healing, and the effect of chitin or chitosan upon these functions.

Cell Type	Function in Wound Healing	Effects of Chitin or Chitosan
Red blood cells	Supportive role in fibrin clot formation.	Chitosan forms a coagulum with red blood cells.
Polymorphonuclear neutrophils (PMN)	Clean wound site of foreign particles and cell debris.	Chitin and chitosan attract PMNs to wound site.
Macrophages	Consume dead cells, attract fibroblasts, support skin and blood vessel replacement and synthesis of the extracellular matrix.	Chitin and chitosan attract macrophages. Chitosan stimulates cytokine production (TGF-β1, PDGF, IL-1).
Fibroblasts	Reformation of the dermis and synthesis of extracellular matrix.	Indirect effect through macrophage cytokines and stimulates IL-8 production.
Keratinocytes	Reformation of epidermis.	Indirect effect through macrophage cytokines.

**Table 3:** Human clinical trials utilizing commercially available and custom-made chitin and chitosan wound treatments, resulting in significant improvements in the healing of ear, nasal, oral, and skin wounds, in addition to treatment of ulcers and serious hemorrhages. Data from [162,163,164,165,166,167,168,169,170,171,172,173,174,175,176,177].

Application	Wound Type	Treatment Utilized	Treatment Constituents	Ref.
Ear	Membrane perforation	Beschitin^®^ W (membrane)	Chitin, unknown	[162]
Hemorrhage	Obstetric hemorrhage	Celox^®^ (powder/gauze)	Chitosan, unknown	[163]
Nasal	Postoperative	Surgi shield^®^ (gel)	8% carboxymethyl chitosan, unknown	[164]
Oral	Aphthous stomatitis	Mouthwash	0.5% chitosan powder, polyacrylic acid, methyl-/propylparaben, glycerin	[165]
		Adhesive film	Chitosan powder, sesame oil	[166]
	Postoperative	HemCon^®^ (dressing)	Chitosan, unknown	[167]
	Tooth extraction	Bexident^®^ Post (gel)	Chitosan, chlorhexidine, allantoin, dexpanthenol	[168]
		Chitohem^®^ (powder)	Chitosan, unknown	[169]
Skin	Diabetic	Medisorb^®^ R (membrane/powder)	Butyric-acetic chitin copolyesters, unknown	[170]
	Postoperative	Membrane	Chitosan only	[171]
		Dressing	Carboxymethyl chitosan, unknown	[172]
	Puncture	HemCon^®^ (dressing)	Chitosan, unknown	[173]
	Superficial	Axiostat^®^ (dressing)	Chitosan, unknown	[174]
		Film	Oligochitosan, glycerol	[175]
Ulcers	Pressure, vascular, diabetic ulcers	Topical gel	2% chitosan powder, acetic acid, regenerated cellulose	[176]
		SEQUA^®^ San Chitosan (dressing)	Chitosan, unknown	[177]
